# Extracellular vesicle-derived miR-320a targets ZC3H12B to inhibit tumorigenesis, invasion, and angiogenesis in ovarian cancer

**DOI:** 10.1007/s12672-021-00437-2

**Published:** 2021-11-17

**Authors:** Yan Huang, Midie Xu, Chuyu Jing, Xiaohua Wu, Xiaojun Chen, Wei Zhang

**Affiliations:** 1 Department of Gynecologic Oncology, Fudan University Shanghai Cancer Center, 270 Dong‑an Road, Shanghai, 200032 China; 2 Department of Pathology, Fudan University Shanghai Cancer Center, Shanghai, 200032 China

**Keywords:** Extracellular vesicles, miR-320a, ZC3H12B, Ovarian cancer, Tumorigenesis

## Abstract

Extracellular vesicles (EVs) play crucial roles in intercellular communication. miRNAs derived from EVs emerge as promising diagnostic indicators and therapeutic targets in a variety of malignancies. Tremendous studies have revealed the function of miRNAs derived from EVs in tumorigenesis, metastasis and other aspects. The mechanism of action of EV-derived miRNAs, however, in ovarian cancer remains largely unknown. In this study, EVs were enriched from the ovarian cancer cell lines. EVs as a whole could promote cell proliferation, invasion and new vasculature formation. However, the down-regulated EV-derived miR-320a was demonstrated to potentially suppress tumorigenesis, metastasis and angiogenesis. Moreover, EV-derived miR-320a has been proved to directly regulate a previously unknown target, ZC3H12B. An unreported role of ZC3H12B in promoting ovarian cancer cell proliferation has been elucidated and miR-320a could mediate the expression of ZC3H12B, thereby inhibiting the downstream response. As for the practical clinic values, lower expression of EV-derived miR-320a correlates with shorter survival period, indicating that EV-derived miR-320a may also serve as a prognostic biomarker in ovarian cancer. This research provides new insight into the molecular mechanism of EV-derived miR-320a in ovarian cancer and may provide new therapeutic and prognostic strategies for ovarian cancer treatment.

**Supplementary Information:**

The online version contains supplementary material available at 10.1007/s12672-021-00437-2.

## Introduction

Regardless of the development of new remedies in cancer treatment, ovarian cancer (OC) remains one of the leading causes of the malignancies in female cancer patients with high relapse rate. A high proportion (59%) of patients diagnosed already have distant metastases. The 5-year survival rate is 48% overall. For those with OC at advanced stage, the rate drops to 29%, compared to 92% for primary disease [1]. Great obstacles for improving the prognosis of OC patients include lack of specific and sensitive diagnostic indicators as well as precision therapeutic options based on the tumor molecular genotypes [2, 3]. Therefore, early diagnosis and prevention of metastasis play pivotal roles in improving prognosis of OC.

Extracellular vesicles (EVs) serve as the carrier for intercellular communication and involved in the biological process of tumorigenesis, metastasis, and relapse [4]. A great number of evidence illustrate that EVs with double-layered membrane contain various biomacromolecules, including proteins, lipids as well as nucleic acids. Both normal and cancerous cells can secret EVs with the size in the range of 50–200 nm [5, 6]. Among them, a type of single stranded RNA, miRNA, plays important roles in regulating gene expression by directly binding to the 3′ untranslated region (3′UTR) [7–9], which involves in different pathological processes, including autophagy [10], ageing [11, 12], glucose metabolism [13, 14], virus infections [15] and cardiovascular diseases [16] As for cancer, miRNAs in EVs mediate tumorigenesis [17], metastasis [18], angiogenesis [19], resistance [20], etc., which are not only considered as diagnostic biomarkers, but also therapeutic targets for further exploration.

Previous studies have revealed the function of miR-320a in some types of cancer. For instance, in breast cancer, cohort with low miR-320a expression levels displayed shorter overall survival periods [21]. Additionally, miR-320a was reported to be a tumor suppressor in gastric cancer, esophageal cancer, and clear cell renal cell carcinoma [22–24]. The clinical value of EV-derived miR-320a in OC, however, has not been elucidated yet.

In this study, we successfully isolated the EVs from cancer cells and plasma samples. The oncogenic role of EVs has been proved by in vitro experiments. Differently expressed miRNAs have been identified by miRNA assay and the most downregulated miR-320a was chosen for further investigation. The expression of EV-derived miR-320a was demonstrated to be downregulated in an OC cell line, ES-2, as compared to normal epithelial ovarian cell line, hosepic. Subsequently, we demonstrated that EV-derived miR-320a exhibited the anticancer activity by targeting a previously unknown target, ZC3H12B. EV-derived miR-320a could bind to ZC3H12B directly and regulate the expression of ZC3H12B, thereby affecting the following cellular process. Lastly, the prognosis result showed the potential of EV-derived miR-320a as a promising prognostic biomarker. Our research sheds light on the new insights into the molecular mechanism of EV-derived miR-320a in OC and may provide new insights in OC treatment and predicting prognosis.

## Material and methods

### Cell lines and cell culture

The normal human epithelial cell line, Hosepic and ovarian cancer cell line, ES-2were obtained from cell bank at the Shanghai Institute of Cell Biology, Shanghai, China, which have been authenticated officially. Hosepic cells were cultured in DMEM (Dulbecco’s Modified Eagle Medium) supplemented with 10% fetal bovine serum (FBS), nonessential amino acids, 1% sodium pyruvate (PS) and antibiotics. ES-2 cells were cultured in Mycoy’s media supplemented with 10% fetal bovine serum (FBS), nonessential amino acids, 1% sodium pyruvate (PS), and antibiotics. All cells were cultured in 37 °C incubator with 5% CO_2_.

### Subjects and sample collection

32 plasma samples from advanced-stage OC patients diagnosed were obtained from the tissue bank of Fudan University Shanghai Cancer Center. All samples were stored at − 80 °C prior to use. Informed consent was obtained from all participants when they were asked to collect samples. The survival data were summarized based on the regular follow-ups. This study (SCCIRB-090371-2 in 2015) was approved by the Ethical Committees of Fudan University Shanghai Cancer Center and performed in concordance to the principles of Declaration of Helsinki.

### Isolation of EVs

For EVs from cells, cell culture supernatant was collected and transferred to a 15 ml centrifuge tube and spined at 3000*g* for 10 min at 4 °C to eliminate cell debris in the sample. Subsequently, the supernatant was added to another tube and centrifuged at 100,000*g* at 4 °C for 6 h. The EVs were pelleted at the bottom. 200 μl of 1 × PBS was added to resuspend the exosome pellet and then transferred to a new 1.5 ml tube. The mixture was centrifuged at 12,000*g* for 2 min at 4 °C and the supernatant containing EVs was kept at − 80 °C for further use. The protein concentration was measured using BCA assays (Thermo Fisher, USA).

### Characterization of EVs

As suggested by the International Society of Extracellular Vesicles (ISEV), a guideline for standardized characterization of particular EVs has been proposed [25], leading to further reproducible information through different studies. In this study, the protein markers and morphologies of the isolated samples were evaluated. The proteins in EVs were dissolved in RIPA buffer. The solution was boiled at 95 °C for 5 min and then the mixture was spined at 10,000*g* for 5 min to obtain the clean protein solution. The solution was loaded onto an SDS-PAGE gel (5% stacking gel, 12% running gel, Bio-Rad). After electrophoresis and transferring, the polyvinylidene fluoride (PVEF) membrane (Bio-Rad, Munich, Germany) was incubated with 0.1% BSA in TBST at room temperature for 1 h. The primary antibody was added onto the membrane and incubated at 4 °C for 12 h. After washing, the secondary antibody was incubated with the membrane at room temperature for another 1 h. The membrane was rinsed for three times. Finally, the membrane was detected under the ChemiDoc XRS imaging system (Bio-Rad, Munich, Germany) using an enhanced chemiluminescence (ECL) system (Thermo Scientific, OR, USA). The concentration and size were measured with nanosight (Malvern, UK) and FEI Tecnai™ T12 electron microscope (Thermo Scientific, OR, USA).

### miRNA microarray analyses

miRNAs were extracted by NanoSep 100 K (Pall Corporation, USA) and de-salted by pushing through an ultracentrifuge tube (Sartorius Stedim Biotech). miRNA ULSTM Labeling Kit (Kreatech Diagnostics, The Netherlands) was used to make the fluorescent labels. The pre-hybridized Mouse miRNA OneArray® v5 (Phalanx Biotech Group, Hsinchu, Taiwan) was used for fluorescent labels detection. The hybridization was kept for 16 h at 37 °C and the non-specific binding was removed by rinsing three times. Then the slide was dried and scanned by an Axon 4000B scanner (Molecular Devices, Sunnyvale, CA, USA). The intensity of Cy5 of every single spot was quantified by GenePix 4.1 software (Molecular Devices). R program (2.12.1) was used for data processing. Data were removed whose flag < 0 within all arrays. The normalization was performed using invariant set normalization method.

### Quantification of miRNA levels by RT-qPCR

cDNA was synthesized using PrimeScript RT Reagent Kit with gDNA Eraser (Takara Bio, Shiga, Japan) based on the instructions without further modifications. The quantification process was conducted using TB Green™ Premix Ex Taq™ (Takara Bio, Shiga, Japan) on ABI QuantStudio Dx (Applied Biosystems, USA). Before the amplification and quantification. Even amount of total RNA and cDNA was added in the experiment. The mix was heated at 95 °C for 30 s, followed by 40 cycles of 95 °C for 3 s, 60 °C for 30 s, and 72 °C for 1 s. The internal standard, U6, was also tested as the reference. The 2^–ΔΔCt^ method was applied for determination of miRNA levels.

### Intracellular uptake of EVs

EVs were enriched from patient plasma as described above. The purified EVs were labeled with PKH26 (Thermo Scientific, OR, USA), a red membrane dye, according to the protocol without further modifications, followed by extensive rinsing to eliminate the residue dye. Cells were incubated with PKH-labeled EVs (10 μg/ml) for 30 min. Cells were then washed three times with 1 × PBS to remove EVs on the surface. Subsequently, 4% paraformaldehyde was added and kept for another 5 min. The EVs inside the cells were visualized by confocal microscopy (Carl Zeiss Inc, Thornwood, NY, USA).

### Preparation of miR-320a mimic/inhibitor loaded EVs

An optimized calcium chloride transfection method has been performed to incorporate the miR-320a mimic or inhibitor into the EVs. In brief, 5 ml of EVs-free media was added in a 60-mm petri dish. 200 pmol miRNA mimic or inhibitor were mixed with 20 μg EVs in 1 × PBS. 0.1 M calcium chloride was added and placed on ice for 30 min. Heat shock was applied at 42 °C for 1 min, and the mixture was kept on ice again for 5 min. For any free miR, 5 μg/ml RNase was added and kept for 30 min at 37 °C.

### Cell viability assay

To characterize the cell proliferation, the cell counting kit-8 (CCK-8) colorimetric assay (DOJINDO Molecular Technologies Inc, Kumamoto, Japan) was utilized. Both cell lines were inoculated in 96-well plates. After overnight incubation, 200 nM of the miR-320a mimic/miRNA control or miR-320a inhibitor/inhibitor control were added to the cells for transfection. Each experiment was conducted in triplicate. Cell viability was characterized at 48 h by measuring the optical intensity of each well at 450 nm by a plate reader.

### Cell migration and invasion assay

Matrigel gel was diluted with serum-free culture medium. 100 μl of diluted gel was added into the upper chamber of the 24-well transwell and then kept at 37 °C for 4 h. The cell concentration was adjusted to 5 × 10^5^ cells/ml and 200 μl cell suspension was added to the upper chamber. As for the lower chamber, 600 μl medium with 5 μg/ml fibronectin was added. For migration assay, the plate was placed at 37 °C for 24 h, while invasion for 48 h. After incubation, the non-invasive cells were wiped off from the upper chamber. The transwell was fixed with methanol for 5 min and dried. 500 μl of 0.1% crystal violet was added to the 24-well plate, the membrane was immersed in the culture medium for 30 min at 37 °C. After being rinsed for three times, the chamber was placed under the microscope for imaging.

### Angiogenesis assay

The Matrigel was put in an ice box in a refrigerator at 4 °C to slowly melt the glue overnight in prior to the experiment. 10 μl Matrigel was added to ibidi angiogenesis slides and covered with the lid. The ibidi angiogenesis slide was put into the 10 cm petri dish and stood for about 30 min for gelation. The concentration of cell suspension was adjusted to 2 × 10^5^ cells/ml. 50 μl of cell mixture was seeded to each well. The image of angiogenesis was regularly recorded and the length of tube, the area, the number of rings, and the intercepts were analyzed, respectively.

### Double luciferase reporter assay

The wild-type and muted binding sites of ZC3H12B 3′UTR were incorporated into the firefly luciferase gene within the psiCHECK-2 vector (Promega), respectively. HEK-293T cells were added into 96-well plates. After incubation for 24 h, 10 ng firefly luciferase reporter plasmid and an equal amount of miR-320a mimic or negative control RNA were added for transfection using Lipofectamine 3000 (Invitrogen, CA, USA). After another 24 h, the luciferase assay kit (Promega, Madison, WI, USA) was used according to the manufacturer’s manual without further modifications.

### Statistical analysis

All data are shown as the means ± SEM. One-way analysis of variance (ANOVA) was used for multiple comparisons. Student’s t test was performed for comparisons between two groups. p < 0.05 was considered to indicate statistical significance (*p < 0.05, **p < 0.01, ***p < 0.001). The statistical analysis was performed using GraphPad Prism® (Version 7).

## Results

### Characterization of EVs in ovarian cancer cell lines

**Fig. 1:**
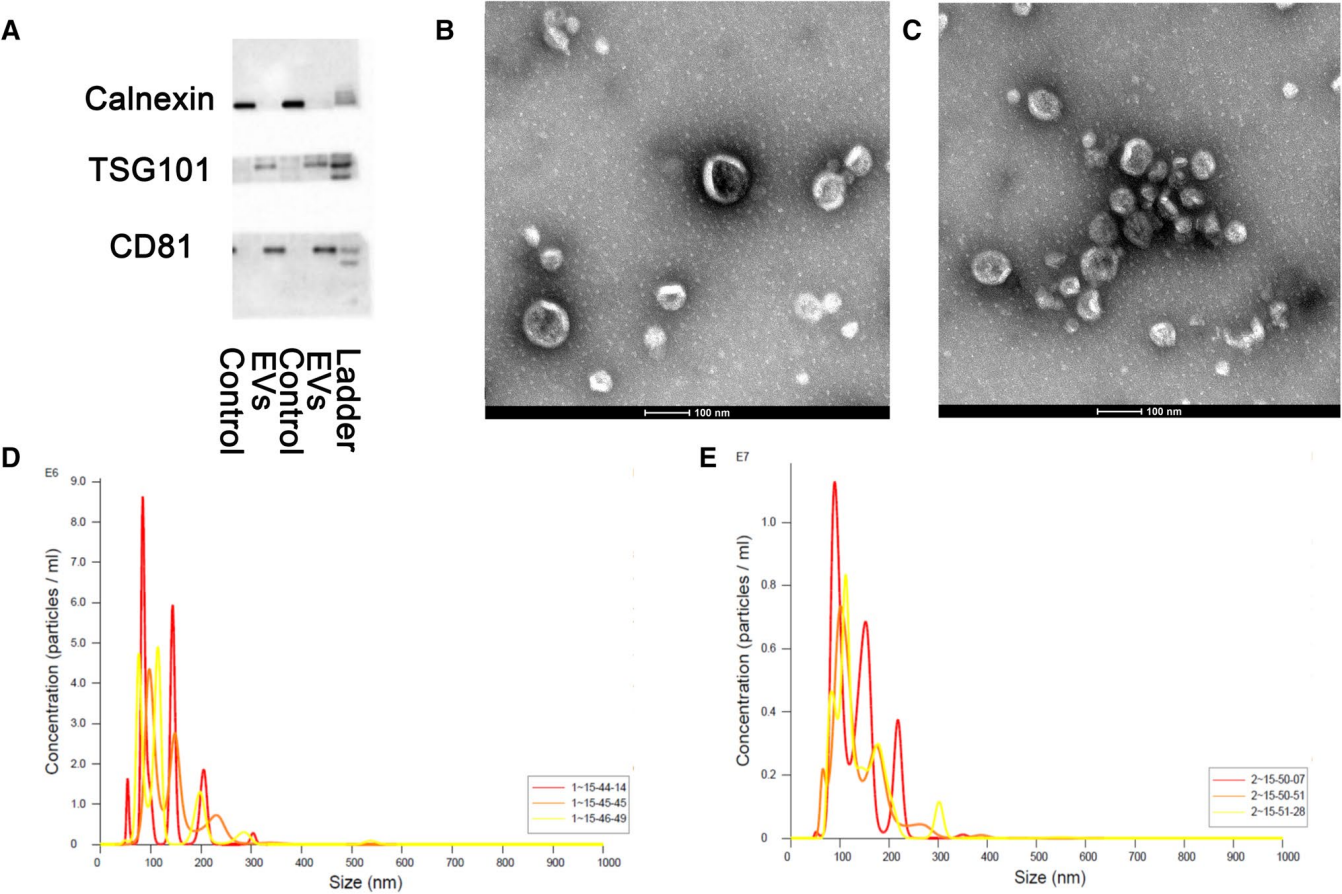
Characterization of EVs. **A** Western blot of typical exosomal markers. **B** TEM of EVs derived from Hosepic cells. **C** TEM of EVs derived from ES-2 cells. **D** NTA of EVs derived from Hosepic cells. **E** NTA of EVs derived from ES-2 cells

CD81 and TSG101 are two typical biomarkers for EVs. Western blotting result showed that the exosome samples contain both markers. No calnexin was detected in EVs samples, indicating the high quality and purity of the isolated EVs (Fig. 1A). Transmission electron microscopy (TEM) was performed to obtain the images of EVs. Figure 1B clearly exhibited the lipid bilayer of the EVs. Additionally, nanoparticle tracking analysis (NTA) was utilized to measure the size and the concentration of EVs, a conventional approach based on the tracking of Brownian movement [26]. The average size measured by NTA was similar to that in TEM characterization (Fig. 1C). In sum, the EVs were successfully isolated from hosepic and ES-2 cell lines with high quality and verified by diverse methods.

### EVs from ES-2 enhances cell proliferation, migration, and invasion and accelerates angiogenesis

**Fig. 2:**
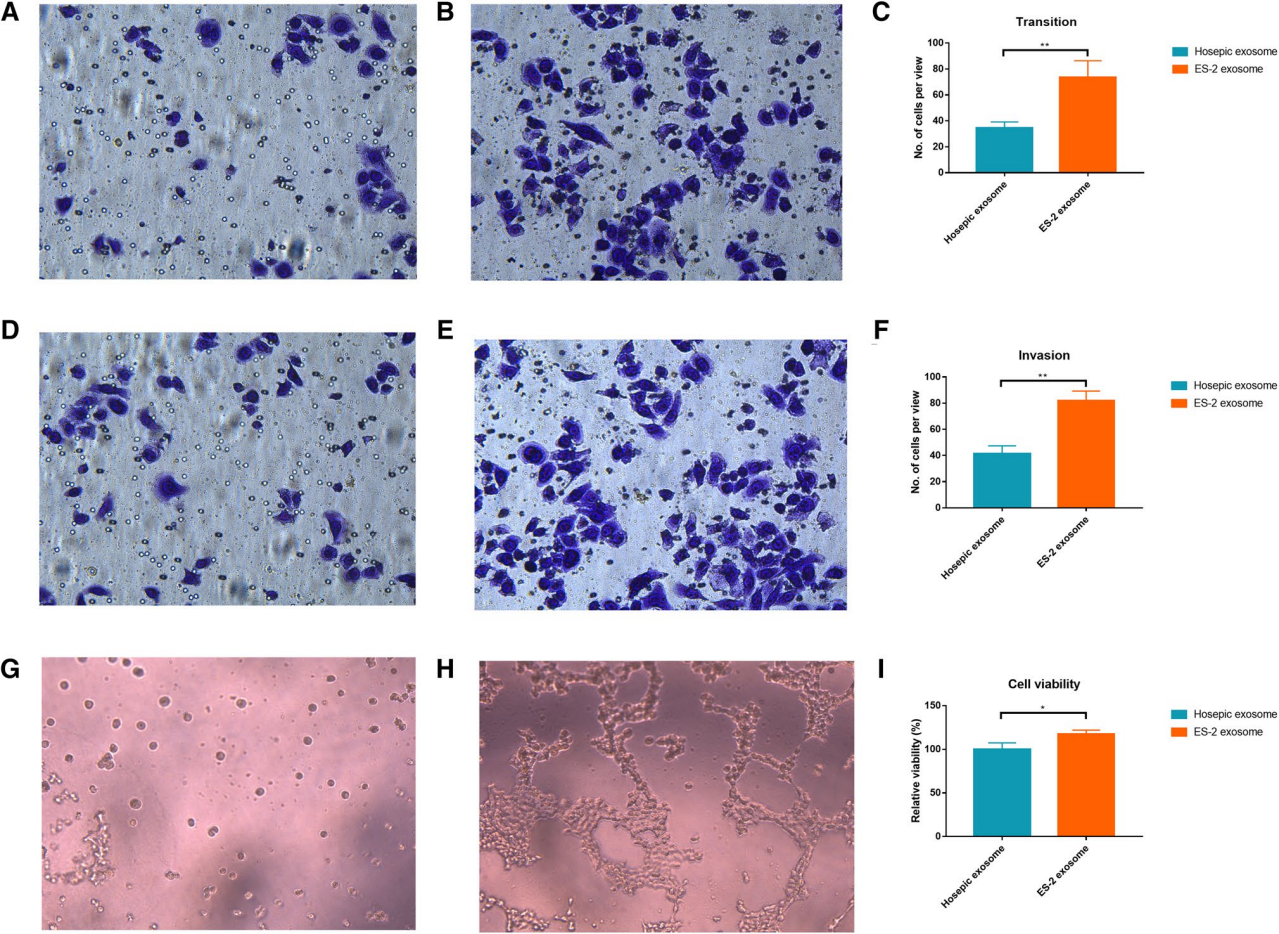
The effect of EVs on cell migration, invasion and angiogenesis. **A** Transition study of Hosepic EVs on Hosepic cells. **B** Transition study of ES-2 EVs on Hosepic cells. **C** Comparison of number of cells per view in transition study. **D** Invasion study of Hosepic EVs on Hosepic cells. **E** Invasion study of ES-2 EVs on Hosepic cells. **F** Comparison of number of cells per view in invasion study. **G** Angiogenesis study of Hosepic EVs on Hosepic cells. **H** Angiogenesis study of ES-2 EVs on Hosepic cells. **I** Cell viability assay of Hosepic and ES-2 EVs on Hosepic cells

In order to investigate the mechanism of action of EVs, the normal epithelial ovary cell line, hosepic, was co-incubated with EVs secreted by hosepic and OC cell line, ES-2. ES-2 EVs remarkably improved the cell viability and promoted the cell proliferation (Fig. 2I). Both migration and invasion assays showed greater cell confluence and higher cell numbers per view (Fig. 2A–F). Moreover, the angiogenesis seemed to be activated and the length and area of tubes were significantly increased with more intercepts between newly formed structure (Fig. 2G, H).

### Differently expressed miRNAs in ES-2 EVs were identified via miRNA array

**Fig. 3:**
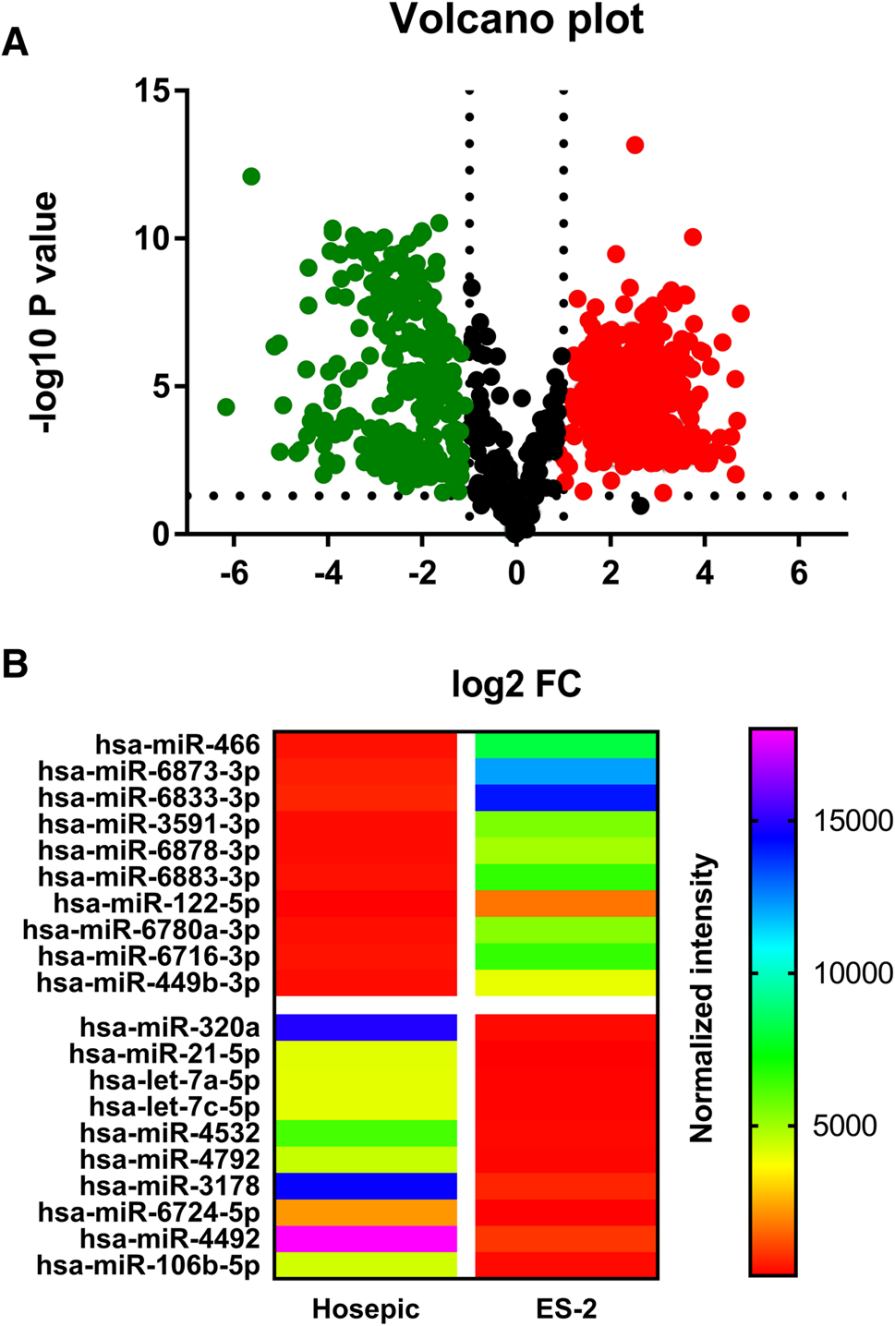
Identification of differently expressed miRNAs. **A** Volcano plot of upregulated and downregulated EV-derived miRNAs. **B** Heatmap of top ten upregulated and downregulated EV-derived miRNAs

The raw EV-derived miRNA array data were normalized and discriminated between ES-2 and hosepic groups. The criterion for screening is that the fold change (FC) has to be within the range of either < 0.5 or > 2. p value must be < 0.05. In total, 486 EV-derived miRNAs were found to upregulated; while another 332 EV-derived miRNAs were downregulated (Fig. 3A). The top ten upregulated and downregulated EV-derived miRNAs were listed in Fig. 3B. Among them, miR-466 is the most upregulated and miR-320a is the most downregulated. Since EVs as a whole could serve as an oncogenic driver, the downregulated miRNAs, such as miR-320a seemed likely to function in an opposite manner.

### miR-320a is confirmed to be downregulated in ES-2 and the corresponding EVs and the transfection of miR-320a mimic enhanced the expression in Hosepic and ES-2 cell lines

**Fig. 4:**
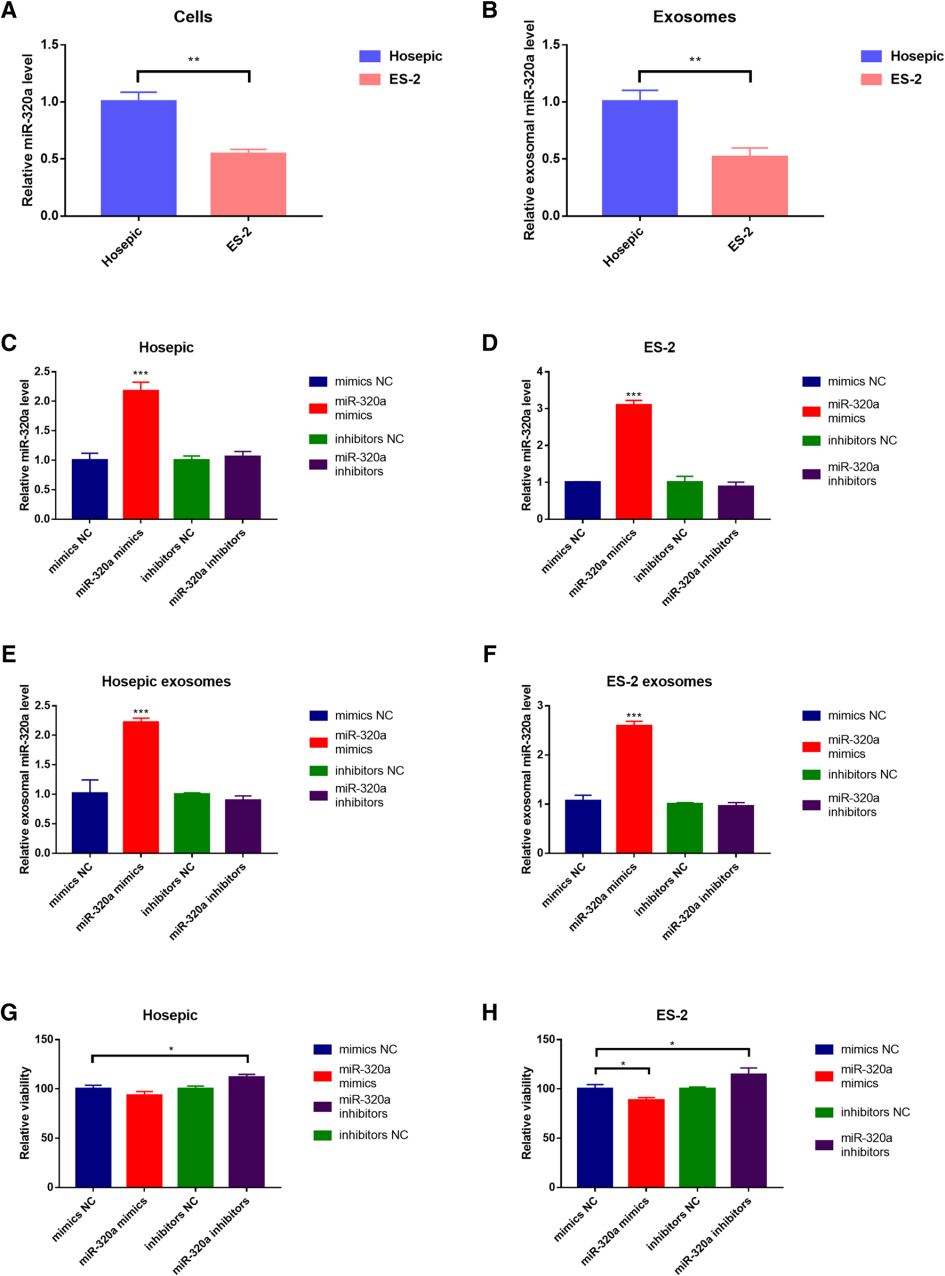
Characterization of miR-320a expression in cells and their corresponding EVs. **A** miR-320a expression in Hosepic and ES-2 cells. **B** miR-320a expression in EVs derived from Hosepic and ES-2 cells. **C** The effect of mimics NC, miR-320a mimics, inhibitors NC, and miR-320a inhibitors on miR-320a expression in Hosepic cells. **D** The effect of mimics NC, miR-320a mimics, inhibitors NC, and miR-320a inhibitors on miR-320a expression in ES-2 cells. **E** The effect of mimics NC, miR-320a mimics, inhibitors NC, and miR-320a inhibitors on miR-320a expression in EVs derived from Hosepic cells. **F** The effect of mimics NC, miR-320a mimics, inhibitors NC, and miR320a inhibitors on miR-320a expression in EVs derived from ES-2 cells. **G** The effect of mimics NC, miR-320a mimics, inhibitors NC, and miR-320a inhibitors on cell viability in Hosepic cells. **H** The effect of mimics NC, miR-320a mimics, inhibitors NC, and miR-320a inhibitors on cell viability in ES-2 cells

The original miR-320a level was quantified in ES-2 and hosepic and their corresponding EVs. Similar to the miRNA array result, the level of miR-320a in ES-2 cells and EVs was significantly lower than that of Hosepic (Fig. 4A, B). Since the EVs were derived from the cells, the contents secreted in the vesicles might not be in proportion to the cellular level. Figure 4C–F showed the results after the miR-320a mimic has been successfully transfected in two cell lines. The mRNA level of miR-320a was verified by RT-qPCR. As expected, the amount of miR-320a dramatically increased as compared to other groups at both cellular and EV levels. Nevertheless, the inhibitor of miR-320a did not affect the expression, which only took effect through binding to the target, blocking the subsequent signaling pathway.

### Visualization of the intracellular uptake of EVs

**Fig. 5:**
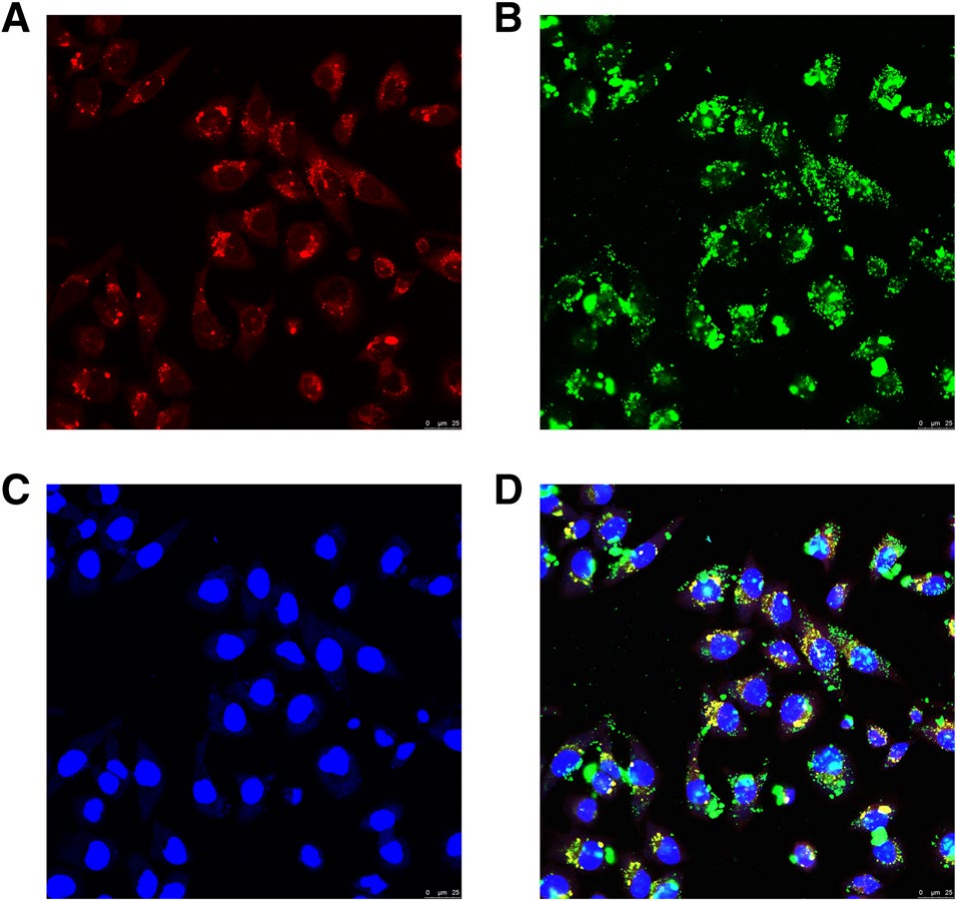
Intracellular uptake of EVs in Hosepic cells. **A** EVs were labeled in red. **B** Cytosols were labeled in green. **C** Nuclei were labeled in blue. **D** The merged image

The intracellular uptake of EVs has been clearly confirmed by the confocal imaging (Fig. 5). Exosome were labeled with a lipid membrane dye, PKH26, in red. The cytosol and nuclei were labeled in green and blue, respectively. The merged image clearly exhibited that the EVs have entered into the recipient cells.

### EV-derived miR-320a attenuates the cell proliferation and suppresses cell migration, invasion and angiogenesis

**Fig. 6:**
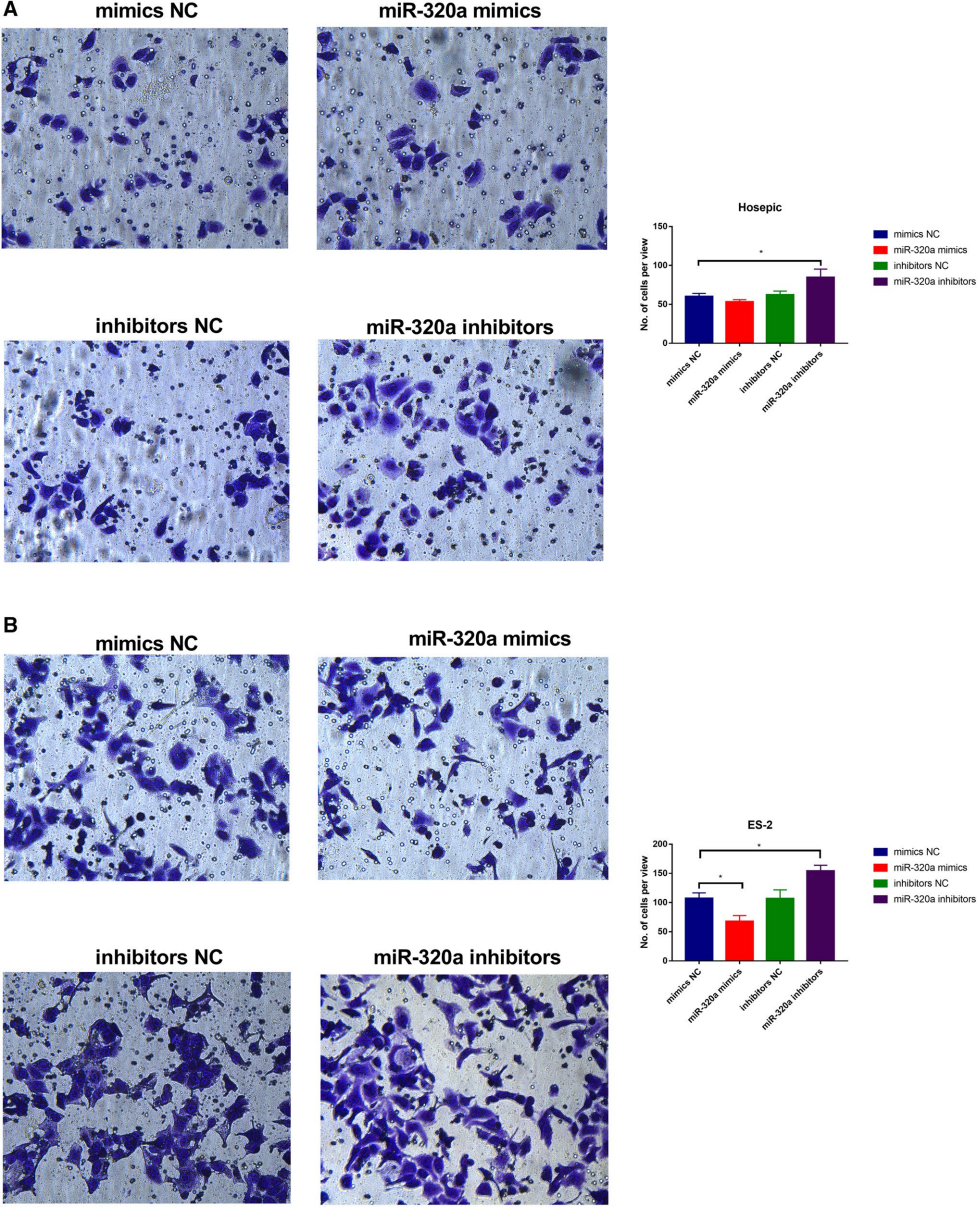
The role of miR-320a in cell migration. **A** Migration study of mimics NC, miR-320a mimics, inhibitors NC, and miR-320a inhibitors in Hosepic cells. **B** Migration study of mimics NC, miR-320a mimics, inhibitors NC, and miR-320a inhibitors in ES-2 cells

Both Hosepic and ES-2 cell lines were treated with miR-320a mimic/inhibitor and control mimic/inhibitor loaded EVs to evaluate their effect on the cell migration, invasion as well as angiogenesis. As shown in Fig. 4G, H, miR-320a distorted the cell viability only in ES-2 group, but not in Hosepic group, implying that overexpressed miR-320 could only inhibit the proliferation in cancer cells. After adding the miR-320a inhibitor, the cell viability of both ES-2 and Hosepic group were elevated. Similarly, miR-320a mimic could crucially suppress cell migration and invasion of ovarian cancer cells, but not in normal ovarian cells. In contrast, miR-320a inhibitor promotes the cell migration and invasion in both cell lines (Fig. 6, Figure S1). Newly generated vessels play a crucial role in supplying nutrients and oxygen into the core of the tumor tissue. miR-320a mimic could dramatically disturb the process of angiogenesis in ES-2, but not in Hosepic. The length and the area of tubes were significantly increased with more intercepts between newly formed structure after treating with miR-320a mimic (Figure S2).

### EV-derived miR-320a targets ZC3H12B and inhibits the expression of ZC3H12B

The 3′UTRs of mRNAs are considered as the targets of miRNAs to regulate gene transcription. RNAhybrid, miRanda and, TargetScan were used to analyze the target genes of miR-320a. Three target genes have been selected based on the overlapped prediction, namely ZC3H12B, CLCN3, and TNRC6B. RT-qPCR was performed to quantify the level of target genes in normal and cancerous ovarian cell lines. Without any interference, all three target genes were highly expressed in ES-2 at both cellular and EV levels (Figure S3A–F). ZC3H12B was the most expressed target gene, which was chosen for further studies. Once the external miR-320a mimic has been incorporated, the expression of ZC3H12B dramatically declined in both Hosepic and ES-2 at cellular and EV level (Figure S3G, H); while after adding miR-320a inhibitor, the expression almost doubled in Hosepic and ES-2 cells and their EVs (Figure S3I, J). To further explore whether ZC3H12B is a direct target of miR-320a, a dual luciferase reporter assay was performed. The intensity of luminescence was measured in different groups. The negative control did not interfere with the luciferase activity. Moreover, miR-320a mimic did not lower the luminescence because of the vector without the 3’UTR sequence of ZC3H12B. Surprisingly, cells transfected with both miR-320a mimic and the vector containing the 3ʹUTR sequence of ZC3H12B displayed remarkably lower luciferase activity among all groups. On the contrary, co-infection of miR-320a with the ZC3H12B 3ʹUTR mutant exhibited no significant change, suggesting that the binding of miR-320a to ZC3H12B is straightforward (Figure S3K).

### Increased expression of target genes of miR320a correlated with poor prognosis

For prognostic evaluation, firstly, the survival of patients was analyzed based on TCGA database. The subjects were classified into 2 groups based on the gene expression level (high vs. low). As can be seen from Figure S4A, B, elevated expression of ZC3H12B (HR = 1.55, p = 0.0089) was associated with shorter survival, reflecting a worse prognosis. Whereas this result ought to be interpreted with caution because these target genes were evaluated on a platform that only consists of several thousand cases. Subsequently, the prognostic potential of EV-derived miR-320a was investigated in OC patients. Plasma samples from 32 OC patients in the sample bank were used for quantification of miR-320a level. The relapse-free survival (RFS) and overall survival (OS) were summarized based on the results of regular follow-ups. Both RFS and OS in high miR-320a group were longer than that in low miR-320a group, indicating a late recurrence and better prognosis (Figure S4C, D). Therefore, EV-derived miR-320a could be a promising prognostic biomarker in OC to predict survival.

## Discussion

The cell communication is mediated by EVs secreted by various types of cells [27]. Apart from the advances in exosome research, the functions of EV-derived miRNAs have also been extensively investigated. miRNAs were transported by EVs to regulate various cellular bioactivities [28–30]. In this study, EVs enriched from cancer cells were proved to enhance the cell growth and invasion. On the contrary, the down regulated EV-derived miR-320a suppressed tumorigenesis, invasion, and angiogenesis of ovarian cancer by directly targeting a novel target, ZC3H12B. Besides, the data from clinical samples revealed that EV-derived miR-320a could also be a prognostic indicator associated with patient outcomes.

Several studies have already studied the function of miR-320a in other types of cancer. For instance, serum miR-320a was proved to directly bind to PBX3 protein in melanoma and inhibit the malignant phenotype of cells and affects the occurrence and progression of melanoma [31]. Another research group explored the function of miR-320a in retinoblastoma tissues. Inhibition of miR-320a alleviates the proliferation and induces apoptosis of retinoblastoma cells through the pathway involving TUSC3 [32]. Additionally, miR-320a could be used as a diagnostic indicator to differentiate tumor and benign tissues with high sensitivity and specificity. Further experiments proved the direct binding between miR-320a and 3ʹUTR of PD-L1 and overexpression of p53 leads to the upregulation of miR-320a, implying that defective p53-regulated miRNA response led to PD-L1 expression and induced immune evasion in malignant pleural mesothelioma [33]. Moreover, the function of EV-derived miR-320a has also been studied. For instance, loss of exosomal miR-320a contributes to the development of hepatocellular carcinoma. Exosomal miR-320a in cancer-associated fibroblasts (CAF) inhibited HCC progression and could be a treatment target for further exploration [34]. Interestingly, similar results were obtained in another study. Lower level of exosomal miR-320a expression in serum reflects poorer prognosis in HCC patients. Besides, decreased exosomal miR-320a was also associated with metastasis in lymph node, vascular invasion, and more severe disease stages [35]. Another group discovered that miR-320a in extracellular vesicles (EVs) distorts endometrial cancer by disrupting the HIF1α/VEGFA signaling pathway. Exosomal miR-320a could mediate the downregulation of HIF1α, resulting in lowered expression of VEGFA. These results suggest exosomes derived from CAF containing elevated miR-320a which could be a novel therapeutic tactic for endometrial cancer [36]. Apart from studying the tumor suppressor mechanism, miR-320a in exosomes has also been proposed as a diagnostic marker. Metastatic and non-metastatic non-small cell lung cancer can be differentiated with exosomal miR-320a. The combination of CEA, miR-320a, miR-622, and Cyfra21-1 had an improved efficiency for the diagnosis of metastatic NSCLC [37]. In endometriosis, exosomal miR-320a and miR-22-3p in serum were significantly increased, as compared to that in benign individuals, indicating the potential for endometriosis diagnosis [38]. Limited research has been conducted on ZC3H12B, especially in oncology. In one study, the elevated level of ZC3H12B distorts tumor progression by trapping the cell cycle in the G2 phase. The mRNA level of ZC3H12B was found to be increased in the neuroblastoma cell line SH-SY5Y and human brain. The specific modulators remain to be furthered discovered [39].

In conclusion, it is the first time to propose the idea that EV-derived miR-320a directly binds to ZC3H12B to exert its antitumor effect in ovarian cancer. However, the function of miR-320a remains to be complicated and may play various roles in different types of diseases, not only in cancer. Our group only performed the in vitro experiments and animal study is planned to be conducted in the near future to verify its function in vivo. Besides, a study illustrated that numerous PKH26 nanoparticles are formed during PKH26 dye staining of exosomes without clear differentiation. Next time the sucrose gradient should be applied to remove PKH26 nanoparticles for uptake studies [40]. The current study demonstrated that EV-derived miR-320a was downregulated in ovarian cancer cells. In vitro studies displayed the suppressor function of miR-320a in tumorigenesis, invasion, and angiogenesis by directly targeting ZC3H12B to attenuate the expression. Lower expression of EV-derived miR-320a and higher expression of ZC3H12B correlate with shorter survival period, indicating that EV-derived miR-320a may also serve as a prognostic biomarker in ovarian cancer. This research provides new insight into the mechanism of EV-derived miR-320a in ovarian cancer and propose new directions for OC treatment.

## Supplementary Information

Supplementary file 1 (TIF 36642 KB). Figure S1. The role of miR-320a in cell invasion. (A) Invasion study of mimics NC, miR-320a mimics, inhibitors NC, and miR-320a inhibitors in Hosepic cells. (B) Invasion study of mimics NC, miR-320a mimics, inhibitors NC, and miR-320a inhibitors in ES-2 cells.

Supplementary file 2 (TIF 6451 KB). Figure S2. The role of miR-320a in angiogenesis. (A) Angiogenesis study of mimics NC, miR-320a mimics, inhibitors NC, and miR-320a inhibitors in Hosepic cells. (B) Angiogenesis study of mimics NC, miR-320a mimics, inhibitors NC, and miR-320a inhibitors in ES-2 cells.

Supplementary file 3 (TIF 835 KB). Figure S3. The expression of miR-320a target gene. (A) The expression of ZC3H12B in Hosepic and ES2 cells. (B) The expression of TNRC6B in Hosepic and ES-2 cells. (C) The expression of CLCN3 in Hosepic and ES-2 cells. (D) The expression of ZC3H12B in EVs derived from Hosepic and ES2 cells. (E) The expression of TNRC6B in EVs derived from Hosepic and ES-2 cells. (F) The expression of CLCN3 in EVs derived from Hosepic and ES-2 cells. (G) The effect of mimics NC, miR-320a mimics, inhibitors NC, and miR-320a inhibitors on ZC3H12B expression in Hosepic cells. (H) The effect of mimics NC, miR-320a mimics, inhibitors NC, and miR-320a inhibitors on ZC3H12B expression in ES-2 cells. (I) The effect of mimics NC, miR-320a mimics, inhibitors NC, and miR-320a inhibitors on ZC3H12B expression in EVs derived from Hosepic cells. (J) The effect of mimics NC, miR-320a mimics, inhibitors NC, and miR-320a inhibitors on ZC3H12B expression in EVs derived from ES-2 cells. (K) Luciferase assay to prove the binding of miR-320a to ZC3H12B.

Supplementary file 4 (TIF 1260 KB). Figure S4. Prognosis analysis of miR-320a and its target gene, ZC3H12B. (A) Expression of ZC3H12B in low and high risk groups based on TCGA data. (B) Survival curve of different expression of ZC3H12B based on TCGA data. (C) Relapse-free survival curve in low and high expression of miR-320a groups. (D) Overall survival curve in low and high expression of miR-320a groups.

## Data Availability

The original data can be shared upon reasonable request.
