# Supplementary figures and images for: Extracellular vesicle-derived miR-320a targets ZC3H12B to inhibit tumorigenesis, invasion, and angiogenesis in ovarian cancer
Source: Discov Oncol. 2021 Nov 17;12:51. doi: 10.1007/s12672-021-00437-2 (PMC8777536; doi:10.1007/s12672-021-00437-2)

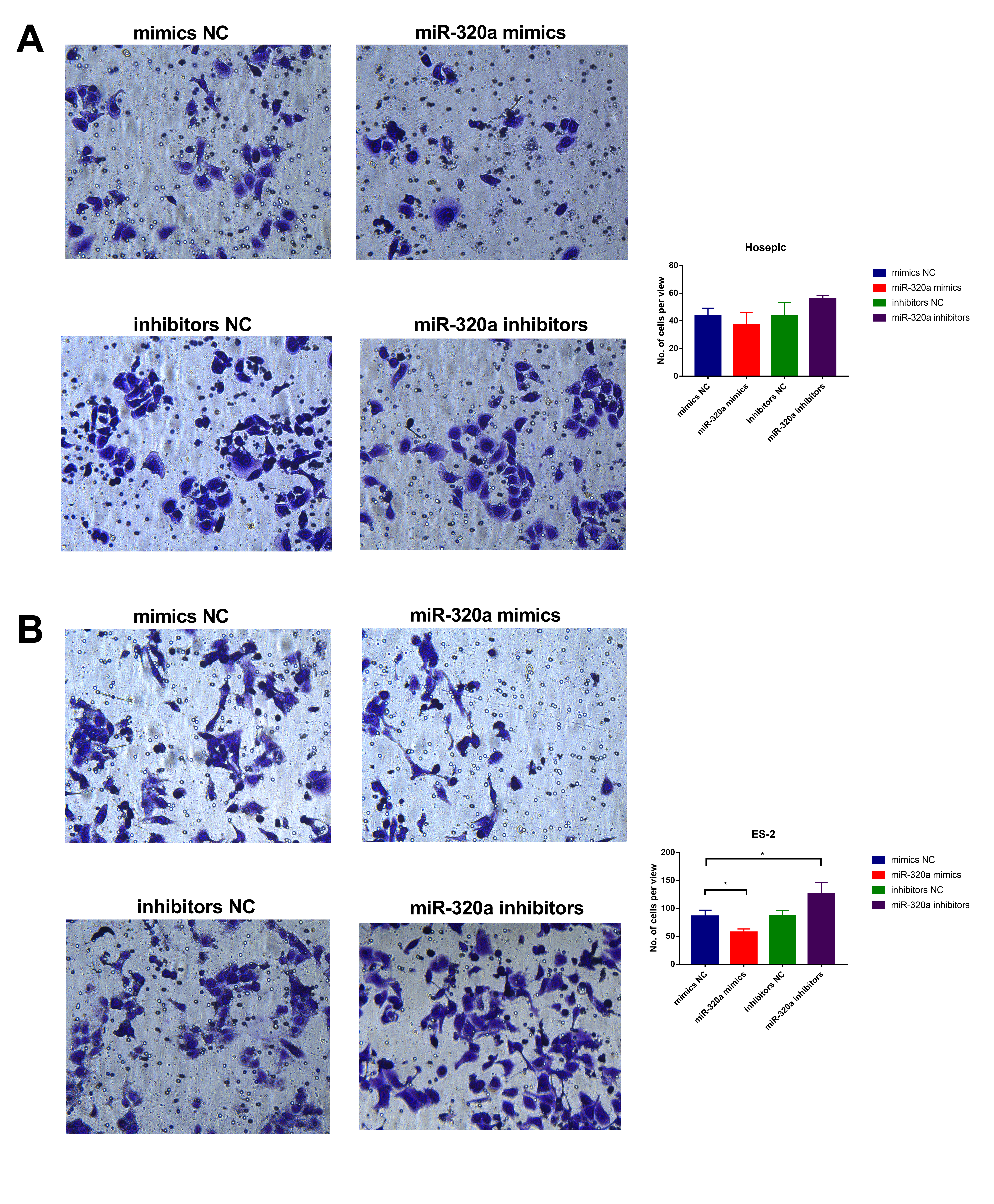

Supplement: Supplementary file 1 — (TIF 36642 KB). Figure S1. The role of miR-320a in cell invasion. (A) Invasion study of mimics NC, miR-320a mimics, inhibitors NC, and miR-320a inhibitors in Hosepic cells. (B) Invasion study of mimics NC, miR-320a mimics, inhibitors NC, and miR-320a inhibitors in ES-2 cells. [file 12672_2021_437_MOESM1_ESM.tif]

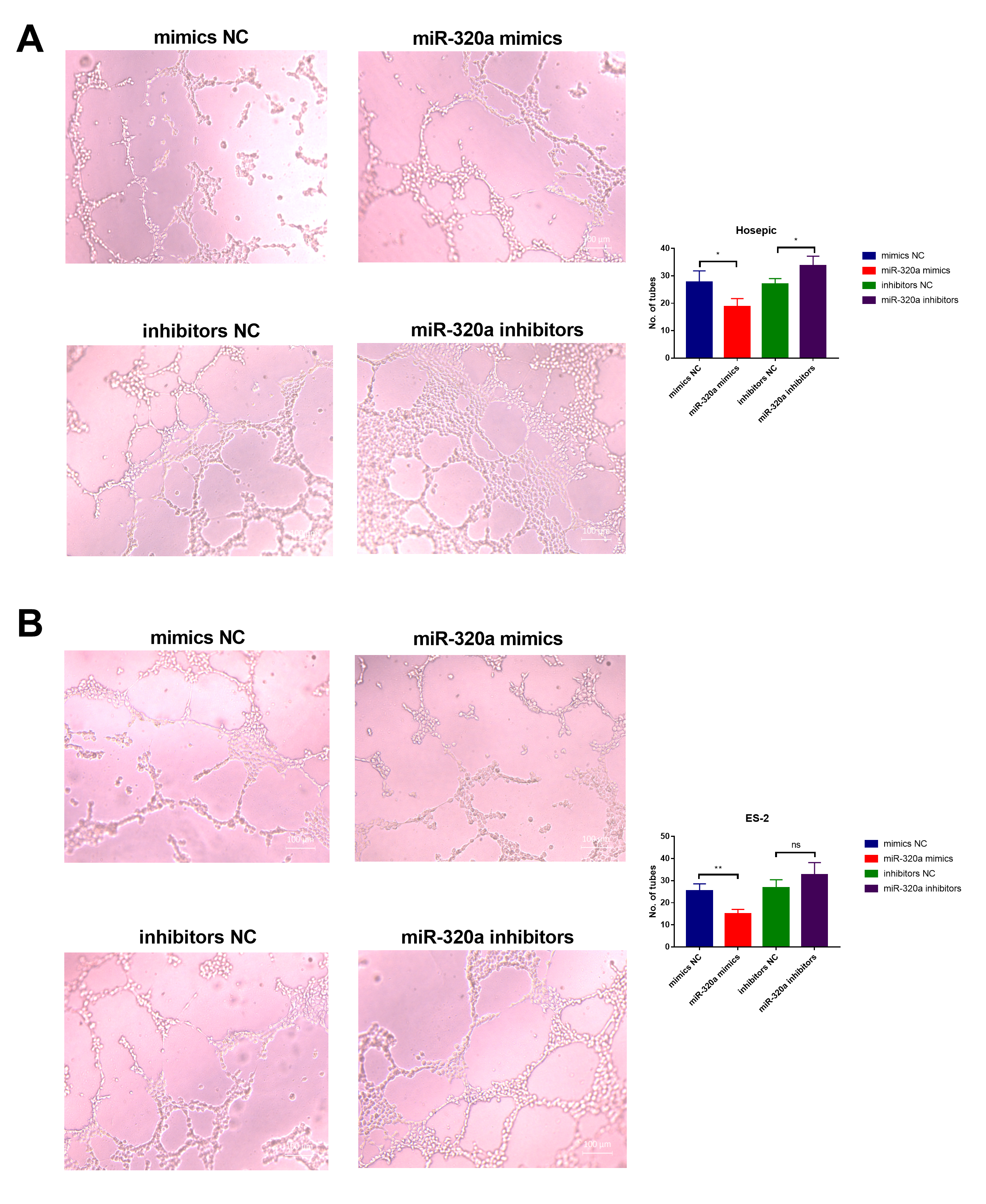

Supplement: Supplementary file 2 — (TIF 6451 KB). Figure S2. The role of miR-320a in angiogenesis. (A) Angiogenesis study of mimics NC, miR-320a mimics, inhibitors NC, and miR-320a inhibitors in Hosepic cells. (B) Angiogenesis study of mimics NC, miR-320a mimics, inhibitors NC, and miR-320a inhibitors in ES-2 cells. [file 12672_2021_437_MOESM2_ESM.tif]

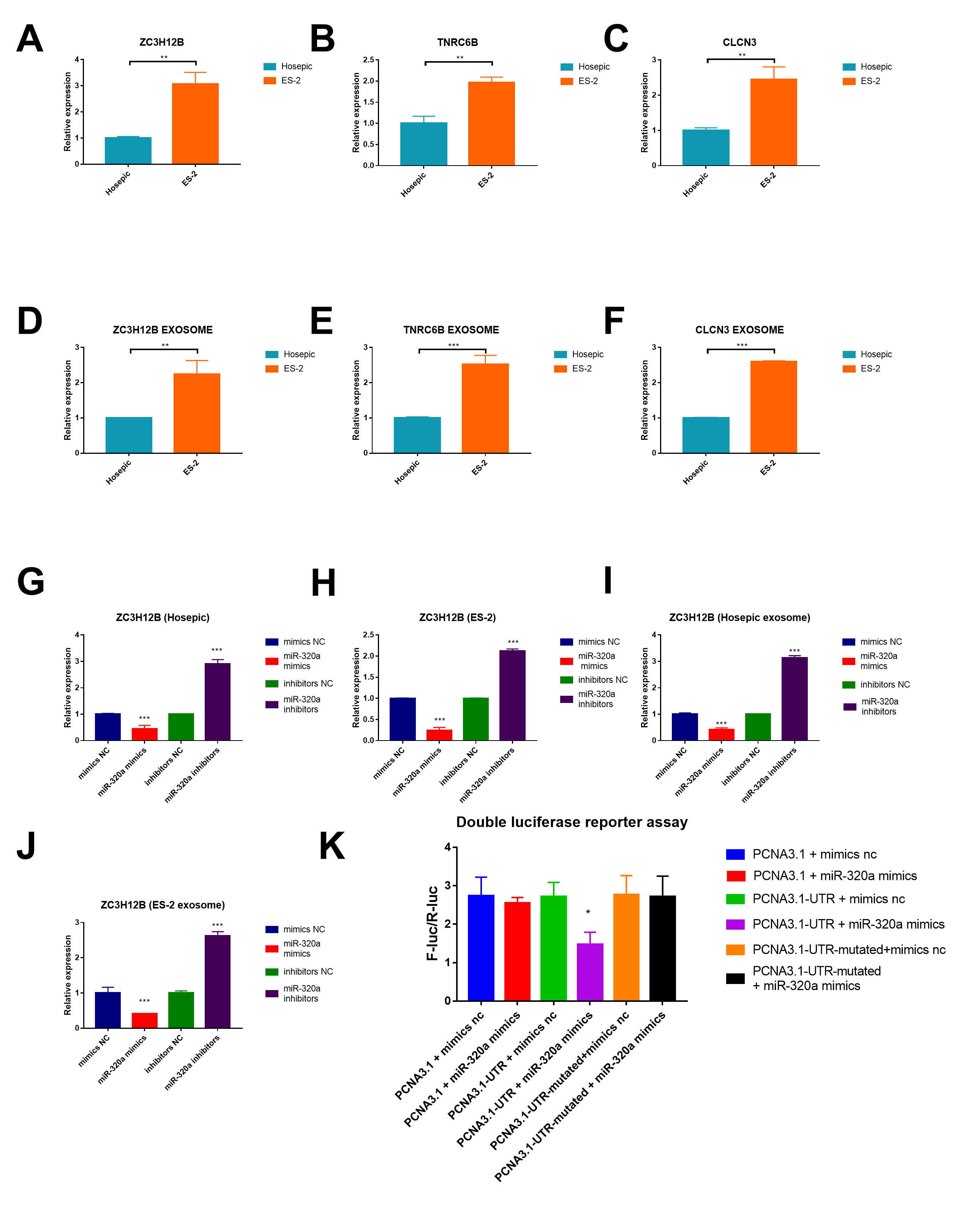

Supplement: Supplementary file 3 — (TIF 835 KB). Figure S3. The expression of miR-320a target gene. (A) The expression of ZC3H12B in Hosepic and ES2 cells. (B) The expression of TNRC6B in Hosepic and ES-2 cells. (C) The expression of CLCN3 in Hosepic and ES-2 cells. (D) The expression of ZC3H12B in EVs derived from Hosepic and ES2 cells. (E) The expression of TNRC6B in EVs derived from Hosepic and ES-2 cells. (F) The expression of CLCN3 in EVs derived from Hosepic and ES-2 cells. (G) The effect of mimics NC, miR-320a mimics, inhibitors NC, and miR-320a inhibitors on ZC3H12B expression in Hosepic cells. (H) The effect of mimics NC, miR-320a mimics, inhibitors NC, and miR-320a inhibitors on ZC3H12B expression in ES-2 cells. (I) The effect of mimics NC, miR-320a mimics, inhibitors NC, and miR-320a inhibitors on ZC3H12B expression in EVs derived from Hosepic cells. (J) The effect of mimics NC, miR-320a mimics, inhibitors NC, and miR-320a inhibitors on ZC3H12B expression in EVs derived from ES-2 cells. (K) Luciferase assay to prove the binding of miR-320a to ZC3H12B. [file 12672_2021_437_MOESM3_ESM.tif]

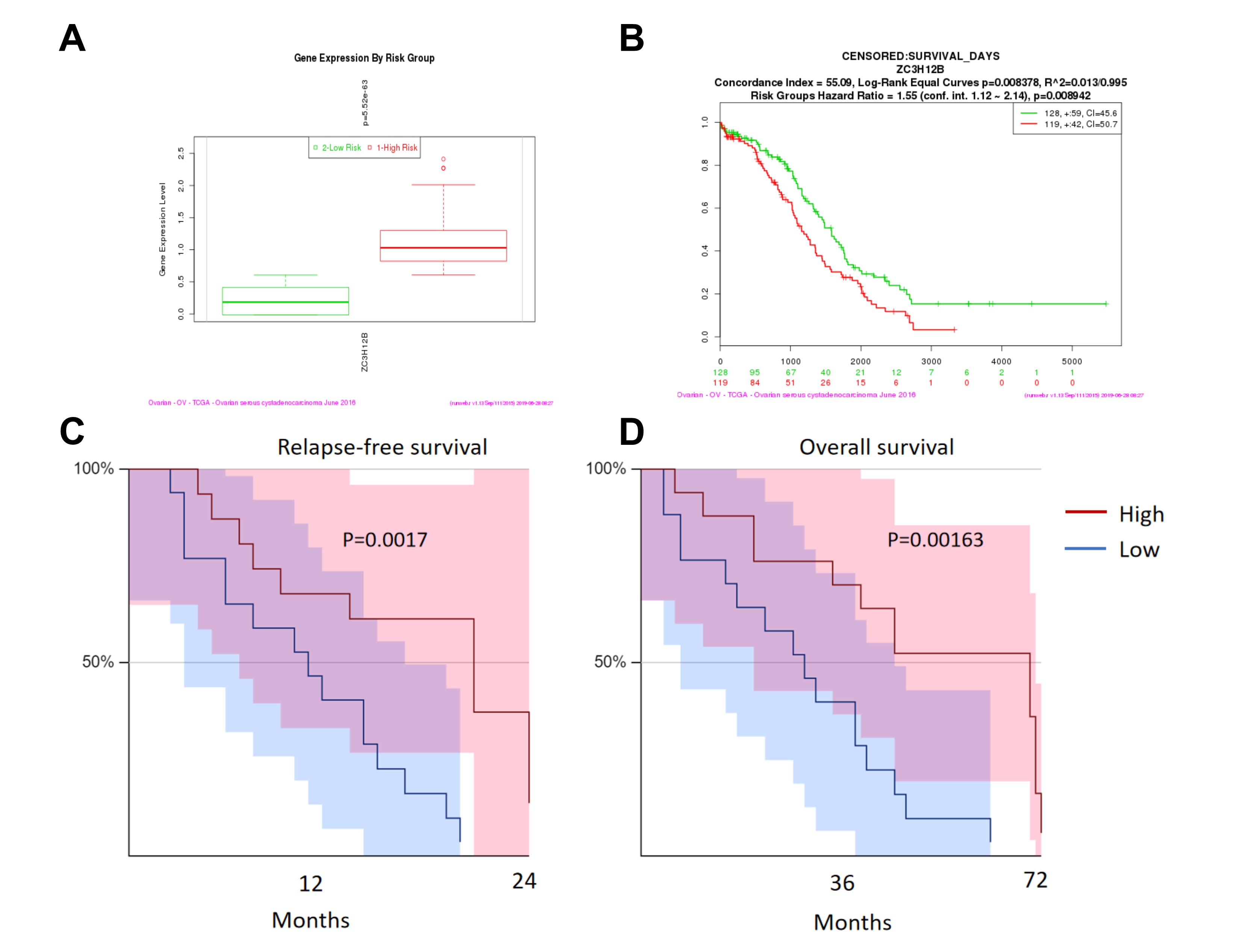

Supplement: Supplementary file 4 — (TIF 1260 KB). Figure S4. Prognosis analysis of miR-320a and its target gene, ZC3H12B. (A) Expression of ZC3H12B in low and high risk groups based on TCGA data. (B) Survival curve of different expression of ZC3H12B based on TCGA data. (C) Relapse-free survival curve in low and high expression of miR-320a groups. (D) Overall survival curve in low and high expression of miR-320a groups. [file 12672_2021_437_MOESM4_ESM.tif]
